# Plant Beneficial Bacteria as Bioprotectants against Wheat and Barley Diseases

**DOI:** 10.3390/jof8060632

**Published:** 2022-06-14

**Authors:** Emma Dutilloy, Feyisara Eyiwumi Oni, Qassim Esmaeel, Christophe Clément, Essaid Ait Barka

**Affiliations:** Université de Reims Champagne-Ardenne, INRAE, RIBP USC 1488, SFR Condorcet, 51100 Reims, France; emma.dutilloy@univ-reims.fr (E.D.); feyisara.oni@univ-reims.fr (F.E.O.); christophe.clement@univ-reims.fr (C.C.); ea.barka@univ-reims.fr (E.A.B.)

**Keywords:** beneficial bacteria, *Triticum aestivum*, *Hordeum vulgare*, phytopathogenic fungi, defense priming, plant immunity, secondary metabolites

## Abstract

Wheat and barley are the main cereal crops cultivated worldwide and serve as staple food for a third of the world’s population. However, due to enormous biotic stresses, the annual production has significantly reduced by 30–70%. Recently, the accelerated use of beneficial bacteria in the control of wheat and barley pathogens has gained prominence. In this review, we synthesized information about beneficial bacteria with demonstrated protection capacity against major barley and wheat pathogens including *Fusarium graminearum*, *Zymoseptoria tritici* and *Pyrenophora teres*. By summarizing the general insights into molecular factors involved in plant-pathogen interactions, we show to an extent, the means by which beneficial bacteria are implicated in plant defense against wheat and barley diseases. On wheat, many *Bacillus* strains predominantly reduced the disease incidence of *F. graminearum* and *Z. tritici*. In contrast, on barley, the efficacy of a few *Pseudomonas*, *Bacillus* and *Paraburkholderia* spp. has been established against *P. teres*. Although several modes of action were described for these strains, we have highlighted the role of *Bacillus* and *Pseudomonas* secondary metabolites in mediating direct antagonism and induced resistance against these pathogens. Furthermore, we advance a need to ascertain the mode of action of beneficial bacteria/molecules to enhance a solution-based crop protection strategy. Moreover, an apparent disjoint exists between numerous experiments that have demonstrated disease-suppressive effects and the translation of these successes to commercial products and applications. Clearly, the field of cereal disease protection leaves a lot to be explored and uncovered.

## 1. Wheat and Barley: Duo Cereals at the Foundation of Global Food Stability

Wheat and barley are among the main cereals cultivated worldwide with an estimated production of 765 million tons and 140 million tons, respectively, in 2020 [1]. The production of these cereals is crucial in view of their global use as both food and animal feed. Thus, both crops are the staple food for a third of the world’s population. The world’s cultivated area remains generally stable, with increasing yields mainly due to genetic selection [2,3].

Wheat is a monocotyledonous crop belonging to the kingdom Plantae, the class Liliopsida, the family Poaceae and the genus *Triticum*. The genus *Triticum* contains approximately 300 species occurring worldwide. Their genome plasticity has allowed them to occur across the globe with more than 25,000 different wheat varieties adapted to a large number of environments [4,5]. *Triticum* has two major species, namely common wheat, *Triticum aestivum* (2n = 42; AABBDD), and durum wheat, *Triticum durum* (2n = 28; AABB). Common wheat is mainly grown in northern and eastern Europe, while durum wheat is more suitable for warm regions. Moreover, based on their composition, they are not used for the same purposes. Common wheat, with a high content of gluten and protein, is used to make flour for bread and biscuit products. In contrast, durum wheat is richer in protein and amino acids with harder albumen, which can be transformed into semolina or pasta [6,7].

Barley, also a monocot, belongs to the same class and family as wheat but is affiliated to the genus *Hordeum*. This genus is categorized into three large species groups of which the most commonly cultivated belongs to the species *Hordeum vulgare* (2n = 14) [8]. Based on the fertility of their spikes, barleys can be classified into two groups comprising 2-row barleys and 6-row barleys [9,10]. Another method of classification is based on crop conditions namely spring barley, winter barley and escourgeon, which includes six-row cyclic-spiked varieties [11]. Barley is mainly used for animal feed but is also processed for the manufacture of alcohol (beer and whisky) or as food, particularly in several regions of the world such as North Africa, the Middle East and Asia, where average and annual consumption varies between 2 and 36 kg per person [8].

Like any cereal, the development cycle of these plants is influenced by seasonal and interannual climate variations and can be divided into three main stages: (i) the vegetative phase including germination with root, leaves and tillers growth until the stem elongation phase; (ii) the reproductive phase corresponding to the period between the tiller and the fertilization characterized in particular by the development of flowers and ears; and (iii) the maturation phase of the grains commencing with the fertilization up to the filling and maturation of the grains [12,13,14].

Since 1970, France has been the leading European producer and exporter of wheat ahead of Germany with five million hectares. The main production basins are Bassin Parisien, Nord-Pas-de-Calais, Centre, Poitou-Charentes and Bourgogne [1]. Common wheat accounts for about 54% of the agricultural area dedicated to cereals compared to only 4% for durum wheat. In addition, France ranks second in Europe for barley production, covering 1.77 million hectares for 11 million tons produced. In France, 1.7 million tons of malting barley were transformed into 1.4 million tons of malt in 2019, allowing the country to become the biggest producer in Europe. The main production areas are the Centre, Bourgogne and Champagne-Ardenne regions [1].

## 2. Main Diseases Affecting Barley and Wheat

During their development cycle, cereals such as wheat and barley are subject to different biotic and abiotic stresses. Among them are fungal diseases that can be extremely deleterious to the plant by attacking different organs including the roots, leaves and ears. The main fungal pathogens can be characterized according to their trophic biology as being biotrophic, necrotrophic and hemibiotrophic [15]. Biotrophic agents establish a long-term association with the host within living plant cells. They are highly specialized and absorb the nutrients present in plant cells without inducing cell death. In wheat and barley, biotrophic diseases are yellow and brown rust (caused by *Puccinia striiformis* f. sp. *tritici* and *Puccinia recondita* f. sp. *tritici*, respectively), powdery mildew (caused by *Blumeria graminis* f. sp. *tritici*.) and covered smut and loose smut (caused by *Ustilago hordei* and *Ustilago nuda* respectively). On the other hand, the necrotrophic agents develop by inducing cell death in tissues allowing them to proliferate for example, the pathogen eyespot (caused by *Tapesia yallundae* and *Tapesia acuformis*). Finally, hemibiotrophs are pathogens having an initial biotrophic cycle followed by a necrotrophic phase. Rhynchosporium (caused by *Rhynchosporium secalis*) and ramularia (caused by *Ramularia collo-cygni*) are specifically barley-associated pathogens. Ultimately, Fusarium head blight, Septoria tritici blotch and Net blotch can be considered to be the most harmful diseases on the cultivation of barley and wheat and are also caused by hemibiotrophic fungi [16,17].

### 2.1. Fusarium Diseases

Fusarium is an important disease that affects all plant organs and causes 30–70% yield losses depending on the severity of the attacks [18]. Besides wheat, Fusarium also affects other plants such as oats (*Avena sativa* L.), barley (*Hordeum vulgare* L.), rice (*Zizania palustris* L.), corn (*Zea mays* L.), and sorghum (*Sorghum bicolor* L.). Infection by the *Fusarium* pathogen may give rise to three distinct types of symptoms: Fusarium seedling blight, resulting in germination losses, Fusarium foot rot causing necrosis of crown tissues, and Fusarium head blight (FHB) attacking the ears [19,20]. The FHB is regarded as the main disease of cereals in Europe because of its significant presence worldwide and represents the fourth major global phytopathogenic fungus [21,22]. The disease is characterized by wilting of the ears and premature senescence. If the infection is early, the kernels are hollow and small, wrinkled and have a whitish color. If the contamination is later, the grains may have continued to fill but are pink with high concentrations of mycotoxins.

Fusarium diseases are associated with two genera of phytopathogenic fungi (*Fusarium* and *Microdochium*) that contain 19 species [23]. Under unfavorable conditions, as in winter, fungi survive as chlamydospores or mycelium in the soil. The fungus may then be present in the form of ascospores (anamorph form), which are mainly primary inoculum, and in the form of macroconidia (the teleomorph phase). Ascospores are released and disseminated by wind while the conidiospores present are spread by the splashing effect of water at the upper stages up to the ear. If the conditions are favorable at the level of the ear, meaning that the humidity is near 100% and the temperature is around 20 °C for a period of 48 to 72 h, the spores can germinate and initiate infection. In addition to environmental conditions, the stage of plant development is a key factor. The period of anthesis corresponds to the critical stage of infection due to the high level of expression of stimulants such as choline and betaine which stimulate the growth of *F. graminearum* and contribute to the infection of the ears by the pathogen [19,24,25,26].

Except for the genus *Microdochium*, the majority of species involved in the incidence of Fusarium head blight are capable of producing a wide range of mycotoxins namely Nivalenol (VIN), Deoxynivalenol (DON), Zearalenone (ZEA), Fumonisines (WUF) [27]. These mycotoxins present a major economic and health dilemma for the cereal industry as numerous studies have demonstrated the capacity of these fusariotoxins to inhibit protein synthesis in eukaryotic cells thereby altering the liver and nervous system of humans and animals [27]. Zearalenone causes hormonal disruption due to its structure, which is similar to estrogen [28]. In addition, DON is a virulence factor in wheat and barley that plays a crucial role in the development of the fungus in the ear. Naturally, a non-mycotoxin-producing *Fusarium* isolate is incapable of infecting other spikelets [29]. It has also been observed that at high concentrations, DON elicits different defense mechanisms including the synthesis of hydrogen peroxide (H_2_O_2_) whose main function is to induce cell death [30].

### 2.2. Septoria tritici Blotch

Septoria tritici blotch (STB) is one the most devasting disease of wheat and barley worldwide. Septoria tritici blotch, caused by the fungus ascomycete *Mycosphaerella graminicola* (teleomorph—sexual form) and *Zymoseptoria tritici* (anamorph—asexual form) is the most widespread disease in Europe, affecting crops particularly in humid climates such as north of France, United Kingdom or Germany. The impact on yield can cause up to 40% of yield loss and depends on both the intensity of the infection and the stage of plant growth at the time of contamination, as seed filling is primarily dependent on photosynthesis by the leaves [31]. The initial symptoms are small chlorotic spots on the leaves. Then, in the mature leaves, lesions develop and are mostly long, narrow, delimited by the leaf veins and contain black or brown fruiting structure named pycnidia. *Zymoseptoria tritici*, belongs to the class Dothideomycetes, of order Botryosphaeriales and family Sphaerioidaceae [32]. The fungus is highly dependent on environmental conditions but possess a high genetic diversity that gives them a high adaptive capacity [33]. The infection of *Z. tritici* usually begins during the fall period as soon as the plant is emerged. Sexually propagated ascospores are considered the primary form of primary inoculum. These spores are produced in the crop residues of the previous crop and contaminate the next crop of the following year. Secondary infection is caused by pycnidiospores carried over a shorter distance by splashing (vertical progression) [34]. Contamination begins with spores adhering to leaves that secrete an extracellular matrix to remain attached to the leaf surface until germination. When environmental conditions become favorable with 85% humidity and temperatures around 20 °C, spore germination commences as early as 2 h post-contamination. Infection begins with the penetration of germ tubes into plant tissues and occurs approximately 24–48 h after inoculation. *Zymoseptoria tritici* is a hemibiotrophic pathogen, which develops slowly in the apoplast without penetrating directly into the host cells and without forming specialized structures such as haustorium. Consequently, during this period of about 9 to 16 days, *Z. tritici* does not induce visible symptoms and seems to survive by assimilating nutrients in solution. The transition from the biotrophic phase to the necrotrophic phase results in the appearance of chlorotic lesions developing into necrotic spots [34,35,36,37].

Wheat can also be affected by the Septoria glume blotch (SGB) which attacks leaves and spikes and is caused by *Phaeosphaeria nodorum* (Muller) (teleomorph) and *Stagonospora nodorum* (Berk.) (anamorph). This pathogen is less prevalent in France but can also have important economic impacts with losses up to 50% and a lower grain quality [38]. The disease can affect all plant parts and results in small dark-brown lesions in the leaves and grayish-white center with a dark-brown periphery.

### 2.3. Net Blotch

Net blotch is the most damaging disease for both winter and spring barley and is present worldwide, especially in humid area. This disease can infect leaves, stems, and kernels. Leaf necrosis causes significant economic losses of up to nearly 50% of final yield loss and reduces seed quality in the absence of control over susceptible varieties [39]. Symptoms on leaves are brown necrotic spots increasing in size to form elliptical or fusiform lesions with sometimes chlorotic lesions [40].

The net blotch disease is caused by the ascomycete fungus *Pyrenophora teres* Drechsler, anamorph *Drechslera teres* (Sacc.) Shoemaker, which exists in two forms: *Pyrenophora teres f. teres* (Ptt) and *P. teres f. maculata* (Ptm). *Pyrenophora teres* belongs to the class of Dothideomycetes and order Pleosporales. Morphologically, Ptt and Ptm are indistinguishable, so their identification is based on the visible symptoms of the plant. The disease caused by Ptt has elongated light brown lesions with dark brown necrotic reticulations while Ptm causes ovoid black lesions that are surrounded by a distinct chlorotic zone [41]. *Pyrenophora teres f. maculata* may negatively impact the quality of the grain thereby reducing its ability to be used in malting [42,43].

*Pyrenophora teres* Drechs. is a hemibiotrophic fungus that is mainly in the necrotrophic form rather than biotrophic [44]. The fungus survives as ascospores in seed or residue from previous cultivation. The pseudothecia are formed after harvest and the ascospores during the winter period until spring according to the climatic conditions. Mature ascospores are dispersed by wind and serve as primary inoculum. Similarly, conidia in previously infected soils can be a source of primary contamination. Infection is most effective if temperatures are around 10–15 °C. After the first infection, the fungus produces a large number of conidia used as secondary inoculum. Sporulation occurs when the humidity reaches 100% and the temperature is between 15 and 25 °C for 10 to 30 h [41,45]. Once implanted, the fungus produces hyphae of greater or lesser length before forming a structure called the appressorium allowing the penetration of the pathogen. A study by Ronen et al. (2019) [39] demonstrated that Ptm is 70% more virulent and showed 20% more necrosis compared to Ptt. In addition, the study also confirmed that, depending on location and environmental conditions, one of the two forms of *P. teres* predominates, with Ptm more common in warm areas (from July to September). This finding would also explain the proliferation of Ptm in new regions whose average temperatures are increasing due to climate change [42,46].

Symptoms caused by *P. teres* are mainly caused by several toxins. Four toxins were successively identified in both Ptt and Ptm namely N-(2-amino-2-carboxyethyl)-aspartic acid, anhydroaspergillomarasmine A, aspergillomarasmine A and aspergillomarasmine B. These toxins belong to the class of marasmins whose main function is chelation of iron ions. They are very sensitive to abiotic stress including light and temperature. In addition to these toxins, four metabolites called pyrenoids A, B, C and D were extracted from *P. teres* without showing any phytotoxicity unlike the isoquinoline, pyrenoline A and B toxins, which are known to be toxic to monocotyledons. These toxins are involved in the development of necrosis (pyrenoid A) and chlorosis (pyrenoids B and C) [44,47,48].

## 3. Current Control Strategies against Pathogens of Barley and Wheat

**Figure 1:**
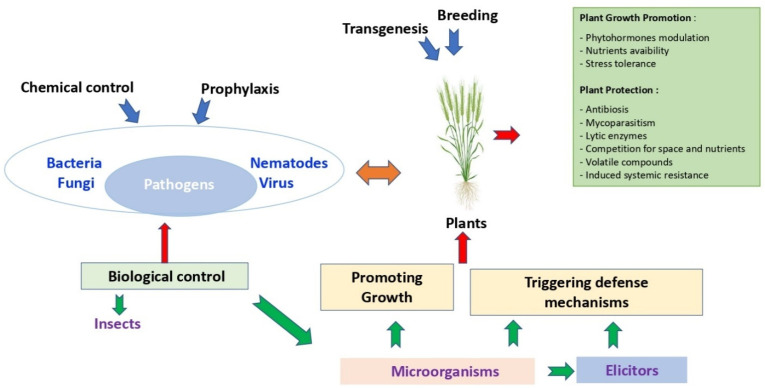
Current strategies of plant protection used in the field. To fight the various biotic stresses and reduce fungal pressure, several strategies are used by the agricultural sector. Chemical control is the most widespread method worldwide because of its effectiveness and its almost systematic use over several generations. However, due to various risks to the environment and human health, other alternatives are increasingly used. Among these alternative solutions, the farmer can rely on various levers such as direct struggles (physical struggle, use of biocontrol, trap plants), indirect control (adaptation of cultivation practices, use of resistant varieties, auxiliaries, microbial ecology).

Besides this strong pathogen pressure, farmers and the various participants in the agricultural sector must also face the emergence of new challenges and expectations from consumers, resulting in changes in consumption patterns and regulations. In order to meet the ever-increasing demand, the stakeholders need to create different control strategies that are mainly based on: chemical, biological, agronomic and genetic factors (Figure 1).

### 3.1. Chemical Control, Prophylactic Strategies and Genetics Selection

Chemical control is the most widespread method worldwide because of its effectiveness and its almost systematic use for several generations. In 2018, more than five million tons of pesticides were used globally to control diseases and pests [1]. At the national level, France is the 3rd largest consumer of pesticides in the world and the 1st in Europe [1]. A wide variety of molecules are already used in marketed products, but their efficiencies depend on the target pathogen. For example, the pathogens *Fusarium* and *Microdochium* do not have the same sensitivity to fungicides. *Fusarium graminearum* is particularly sensitive to triazoles while *F. avenaceum* is more sensitive to strobilurins. Tetraconazole has interesting effects on *F. graminearum* reduction and DON production [49]. Two triazoles, prothioconazole and tebuconazole, show significant efficacy on *Fusarium* with the advantage that prothioconazole is effective on both *Fusarium* and *Microdochium*. The addition of strobilurine, can disrupt the competition between fungi within the ear and cause a reduction of DON. Against Septoria tritici blotch, there are many fungicides such as quinone outside inhibitors (QoI or strobilurins), sterol biosynthesis inhibitors and succinate dehydrogenase inhibitors (SDHI) [50]. The best efficacy is achieved with the combination of a triazole, such as prothioconazole, with one or two active ingredients of the SDHI family such as bixafen in the last leaf stage. However, due to the plasticity of their genome, increasing numbers of species have developed resistance, which can potentially limit the effectiveness of plant protection products. For Net blotch, a study of the in vitro action of several fungicides on *P. teres* shows that the Ptt form is more sensitive than the Ptm form. The strobilurins have been largely used in recent years and contact fungicides are most effective in limiting conidia germination, whereas systemic fungicides show better efficacy on mycelial growth. On the other hand, seed treatments are not very effective on pathogens, particularly in the case of Ptm [51].

Although great progress has been made and limitations were created, the use of a so-called “conventional” agricultural system remains the most immediate and easiest way to prevent disease development. However, intensive use of plant protection products generates several risks: pathogen resistance development, groundwater and soil contamination, wildlife toxicity, trace elements in consumer products, etc. With the awareness of these issues, several international initiatives with agreements and regulatory frameworks to control the use of plant protection products and fertilizers have been established. Among these alternative solutions, the farmer can act on different levers such as direct control (physical struggle, use of biocontrol, trap plants) and indirect control (adaptation of cultural practices, use of resistant varieties, auxiliaries, and microbial ecology). All these control levers must consider different climatic, physiological and agronomic factors. The environmental conditions necessary for the survival and proliferation of pathogens can be partially controlled in the field by the farmer through different cropping practices [52,53]. At the soil preparation stage, tillage management allows the burial of residues and thus limits the risks of disease appearance [52,54,55]. Certainly, primary inoculum of pathogens such as *F. graminearum*, *Z. tritici* or *P. teres* are present in crop residues. The pathogens can survive for several years in the soil but can only develop in the first centimeters of the residue. To confirm this approach, Pfender and Wootke (1988), demonstrated that *P. teres* has a better chance of survival in upper mulch than in buried straw [54].

Secondly, crop rotation involves alternation of crops and lengthening of rotations to break the disease cycle. For instance, the risk of Fusarium Head Blight on wheat is reduced if the previous crop was not wheat, barley or corn but instead another family such as a Fabaceae (soybean) because the main *Fusarium* species of soybeans is *F. sporotrichioides* whereas in wheat it is *F. graminearum* [56]. In some cases, farmers also grow intermediate crops that can trap ascospores in residues and thus prevent them from being spread by wind to other fields [55,57]. A third prophylactic strategy is the management of seed sowing dates. If the date of sowing and thus of flowering coincides with the release of the spores, then the infection is more frequent and more severe. More so, early varieties are generally more resistant to diseases than other slow-growing varieties and are therefore more susceptible to pathogens over a longer time [57,58]. Furthermore, irrigation alters the microclimate of the plot by increasing moisture, resulting in favorable conditions for the pathogen. Depending on the type of irrigation (cannon, sprinkler, ramp), a massive supply of water over a short period increases the risk of leaching of contact chemical products. Besides, depending on the source of water, water may be a vector for some pathogens such as *Fusarium*. Lastly, the concentration of minerals in the soil can have a strong impact on pathogen development. This impact is very dependent on the stage of the plant and the balance between the different elements present in the soil. For example, excess nitrogen with a potassium deficiency causes the activation of enzymes such as amylase, protease and glucosidases in pathogens that promote their development [26]. In addition, the composition of fertilizers can have a profound effect on microbial communities. Organic fertilizers, produced naturally, promote soil processes, and improve soil microbial biomass. This higher microbial biomass supports microbial competition and prevents the proliferation of a small number of micro-organisms including pathogens [59]. However, other studies have shown that pathogens survive in organic fertilizers increasing the risk of contamination [60].

As a complement to the different farming practices, genetic control through using resistant varieties is a widely used method to effectively control wheat and barley diseases. A plant’s resistance is its ability to prevent the infection or growth of a pathogen in its tissues. This resistance is acquired through two main types of resistance: qualitative, with the «gene-for-gene» model of Flor (1971) [61] and quantitative constituting a polygenic resistance. Several genes of specific resistance have been well characterized to describe the wheat-*Z. tritici* interaction and have been called stb1 to stb18 [62]. This kind of resistance is very effective because it enables the activation of the plant’s defense mechanisms but is very often circumvented by the pathogen. Currently, TE 9111 is the most resistant strain in Europe because it has several qualitative resistance genes, namely *Stb11*, *Stb6* and *Stb7* [63,64]. In barley, several major effect QTLs were identified for resistance to *P. teres* on chromosomes 2H [65], 4H [66], 5H [67], 6H [66], and 7H [65]. Clare et al. (2019) [47] characterized a large number of resistance QTLs based on the pathogen Ptt or Ptm. Several genes have a particular effect on the Rpt5 locus which is considered as an important locus in the Ptt-barley interaction. Another gene involved in the *P. teres*-barley interaction is the *HvS40* gene whose expression is induced by jasmonic acid and salicylic acid. Krupinska et al. (2002) [68] observed high expression levels only in leaf tissue with necrosis and chlorosis after infection suggesting that this gene has an important role during infection.

### 3.2. Biological Control

Biocontrol has long been considered as a less efficient, more expensive, and more burdensome method, and has remained mainly used for more specialized crops such as horticulture or market gardening. However, in recent years, with the increase of regulations, biological control including the use of plant growth promoting bacteria (PGPB) or their natural compounds have been extensively studied due to their potential benefits to reduce the use of chemical plant protection products. Subsequently, more companies embrace the potentiality of these products and are actively developing microbe-based product portfolios to suppress cereal diseases (Table 1). 

Thus, combined with optimization of agronomic levers, the use of biocontrol is beginning to find its place in culture systems. The PGPB can directly benefit host plants by improving the absorption of plant nutrients and/or by modulating growth and phytohormones related to stress thereby conferring an evolutionary advantage to the plant [69]. In a field experiment, *Pseudomonas chlororaphis* MA32 was demonstrated to reduce the incidence of several wheat and barley pathogens via promotion of root and shoot growth. The two commercialized products based on this bacterium, Cedomon^®^ and Cerall^®^, are currently largely used in ecological farms in Europe [70].

Indirectly, beneficial bacteria can improve plant health via competition for ecological niches, antibiotic production, lytic enzymes or volatile compounds, or induction of resistance mechanisms in the plant (Table 1) [69,71,72]. These modes of action are particularly studied for the research of new potential marketed bacteria. As demonstrated in Table 1, most of the existing products are composed of bacteria directly impacting the integrity of the pathogens. The Inateq^TM^ Active manufactured by Corteva is based on the properties of the *Streptomyces* sp. 517-02. This strain shows good crop safety and disease control on *Z. tritici* by inhibiting spore germination and also inhibiting mycelial growth post-germination [73]. The production of metabolites and the competition for space and nutrients are also a major part of the mechanisms for biocontrol products as it is the case with the Cerall product [70] or with the *Streptomyces* sp. K61 derivative Mycostop^®^ [74]

## 4. The Context of Plant Defense

**Figure 2:**
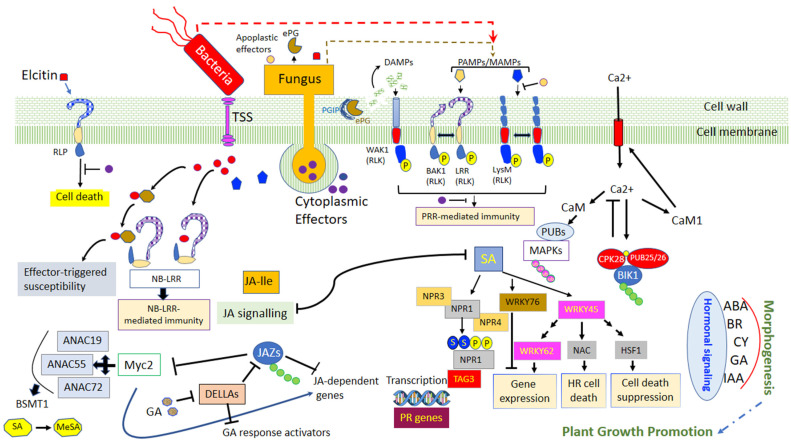
Insights into some molecular factors in plant-pathogen interactions that may be involved in plant immunity. The perception of host damaged-associated molecular patterns (DAMPs), resulting from the interaction between endo-polygalacturonases (ePGs) secreted by some fungi and the PG-inhibiting proteins (PGIPs) secreted by the plant, through DAMP receptors (such as, DAMP receptor wall-associated receptor kinase 1 (WAK1)) triggers plant defense responses. Additionally, microbe-associated molecular patterns (MAMPs) are delivered from microbes to the apoplast (apoplastic effectors) or delivered inside host cells (cytoplasmic effectors) to perturb plant cell physiology. The MAMPs may be perceived by the cell surface pattern-recognition receptors (PRRs; receptor-like kinases (RLKs) or receptor-like proteins (RLPs)) and triggers downstream phosphorylation cascades and provoke an enhancement of [Ca^2+^] and reactive oxygen species (ROS). The activation of pathogen-responsive MAPK cascades is one of the earliest signaling events in PTI and ETI. The pathogen effectors are recognized by intracellular receptors, nucleotide binding site-leucine-rich-repeat (NLRs also known as NB-LRRs), triggering therefore downstream responses including Salicylic acid (SA) accumulation. The results of defense signaling involve modulation of gene expression, the synthesis of (PR) proteins, and biosynthesis of antimicrobial metabolites. More details in the main text. Ub, Ubiquitin. P, phosphate group. S, Small ubiquitin-like modifiers; ABA, Abscisic acid; BR, Brassinosteroid; CAM, Calmodulin; CPK, Calcium-dependent Protein Kinases; CY, Cytokinins; GA, Gibberellic acid; HR, Hypersensitive response; IAA, Indol acetic acid; JA-Ile, Jasmonoyl–isoleucine; JAZ, Jasmonate-zim-domain protein; LysM-RLK, Lysin motif receptor-like kinases; MAPKs, Mitogen-activated protein kinase; NPR, Nonexpressor of pathogenesis-related genes; PUB, Plant U-box; T3SS, type III secretion system.

In any season, plants are confronted with permanent attacks by pathogens, possessing diverse life strategies. Some of these pathogens proliferate outside of the plant tissues, while others can directly penetrate plant cells. The apoplast is the place of the first interaction, between the plants and the pathogens, which is mediated by the recognition of microbial elicitors known as pathogen-associated molecular patterns (PAMPs) and that are identified in plants by membrane-localized pattern recognition receptors (PRRs). To disrupt cellular functions throughout the entire process of infection, pathogens secrete several effectors. In contrast to PAMPs, effectors are various and can involve proteins, chemicals, toxins, or hormones, which elevate pathogen infectiveness by either profiting the pathogen or by suppressing host defenses. Intracellular receptors, termed nucleotide-binding domain, leucine-rich repeat-containing proteins (NLRs, also identified as NB-LRRs), perceive specific effectors carried within the plant cell to activate effector-triggered immunity (ETI). The subsequent recognition of microbial derived PAMPs by PRRs of the plants, which are receptor-like kinases will trigger the first line of defense, called PAMP-triggered immunity (PTI). Among the prompt responses is an extracellular Ca^2+^ influx into the cytosol, which is then succeeded by the induction of cell oxidative burst with the production of the reactive oxygen species (ROS) and the activation of the mitogen-activated protein kinase, and additional signaling molecules, such as reactive nitrogen species, callose, n-hydroxypipecolic acid, salicylic acid, jasmonic acid, ethylene, and cytokinin (Figure 2) [50,75]. 

The synthesis of salicylic acid (SA) follows two different pathways, one involving the phenylalanine (Phe) ammonia-lyase (PAL) and the other, isochorismate synthase 1 (ICS1) [76]. Salicylic acid plays a crucial role in the long-distance signaling mechanism for systemic acquired resistance (SAR) induction, which leads to the localized programmed cell death and the activation of the pathogenesis-related (PR) genes especially the PR1, PR2 and PR5 [77,78]. The signaling of SAR involves lipid transfer protein DIR1 (Defective in Induced Resistance 1) but also different metabolites such as MeSA (Methyl Ester of SA), G3P (Glycerol-3-Phosphate), DA (DiterpenoiddehydroAbietinal), pipecolic acid (Pip) and azelaic acid (AzA) [79,80]. One of the key components for the mediation of the SAR pathway is the protein NPR1 which is a redox-mediated protein used as a transcriptional co-activator of *PR* genes. The *NPR1* gene is a receptor for SA inducing a modification of the protein structure which is essential for the activation of the PR genes [81,82,83]. Furthermore, previous studies have highlighted the existence of two to six *NPR1*-like genes and especially two paralogs of *NPR1*, *NPR3* and *NPR4* that have a different affinity with SA [84]. They regulate the activity and the stability of *NPR1* in different ways: at high SA concentration, *NPR3* supposedly regulates the degradation of *NPR1* during the effector-triggered immunity (ETI) phase resulting in localized programmed cell death whereas *NPR4* is activated with lower SA concentration and causes the activation of *PR* gene expression. In non-stress conditions, *NPR1* is present in quantity as a cytoplasmic oligomer. After an oxidative burst induced by the SA, *NPR1* is monomerized and translocated to the nucleus via a bipartite nuclear localization signal (NLS) to indirectly switch on the PR gene expression via the activation of the TGA family of basic domain/leucine zipper (bZIP) transcription factors. In addition, some WRKY transcription factor genes have been identified to be SA-dependent and potentially involved in the regulation of the expression of *NPR1* [84].

## 5. First Steps of Interaction between Plants and Beneficial Bacteria

### 5.1. Bacteria Perception

**Figure 3:**
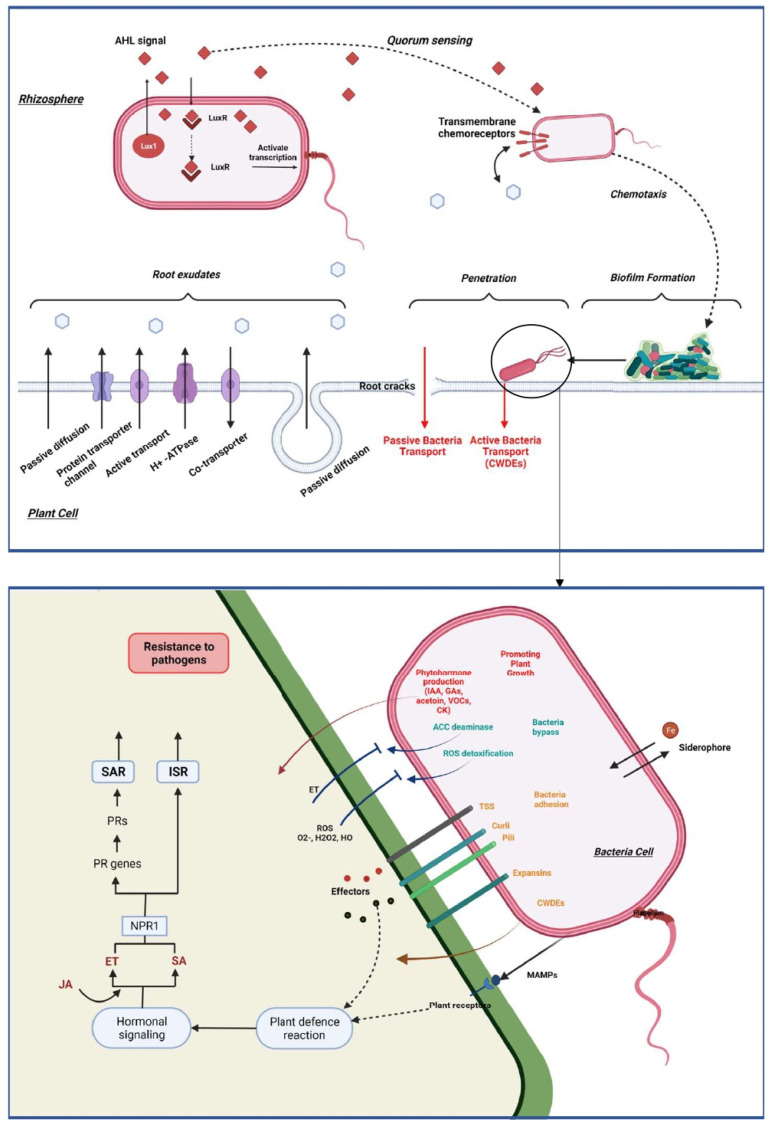
Schematic of plant-bacteria interactions. Abbreviations used in the figure: reactive oxygen species (ROS), oxide anion (O2), hydrogen peroxide (H2O2), type III secretion system (T3SS), type VI secretion system (T6SS), microbe-associated molecular patterns (MAMPs), jasmonic acid (JA), ethylene (ET), salicylic acid (SA), pathogenesis-related proteins (PRs), the nonexpressor of pathogenesis-related gene 1 (NPR1), induced systemic resistance (ISR) and systemic acquired resistance (SAR).

Plants are in constant interaction with other organisms like bacteria, which can interact directly with the surface of the plant and, in some cases, penetrate the tissues and colonize the interspatial region between plant cells. In order to be of benefit to the plant, it is necessary to set up a communication route in the bacteria-plant and bacteria-bacteria directions [71,85] (Figure 3).

For this purpose, specific communication is established between the plant and these bacteria via exudates secretion and the recognition of these compounds through a phenomenon named chemotaxis. These exudates are secreted by the plant root in the rhizosphere and their composition is dependent on plant specificity and environmental factors [86]. The root exudation is mainly passive through diffusion, ion channels or vesicle transport [69]. Plants secrete carbohydrates such as sugar, via an anion channel, metals through metal transporters, whereas water and uncharged molecules are secreted through aquaporins. Additionally, low molecular weight compounds and high molecular weight compounds, are secreted through diffusion and vesicles, respectively. When bacteria perceive the concentration gradient of root exudates, they become motile by means of flagellum or their pili in the specific direction [87,88].

Quorum sensing (QS) has also been studied for its role as a target for host recognition and its implication in communication and recognition during bacteria interactions. Bacteria can synthesize low molecular weight molecules which can be released extracellularly and recognized by autoinducers of other bacteria. Once the extracellular level of the autoinducers attains a critical level, the autoinducers bind to a cellular receptor and trigger a signal transduction cascade. The direct consequence is a change in bacterial gene expression facilitating interaction between cells and enabling the coordination of bacteria through synchronized gene expression [69,89,90]. The most studied signal molecule produced by Gram-negative bacteria is the acyl-homoserine lactone (AHL) which can bind the receptor LuxR-like protein to form a complex which in turn affects gene expression and activate transcription of QS-target genes [90,91,92,93]. This recognition is a key component in the communication between the beneficial bacteria. Indeed, N-AHLs mutants of *Paraburkholderia phytofirmans* PsJN could not colonize efficiently *Arabidopsis thaliana* and promote plant growth [94]. In Gram-positive bacteria, the autoinducers are generally peptides that interact with cognate regulators, phosphatases, or transcriptional regulators. In some PGPR, QS can regulate gene expression in the plant by the induction of plant systemic resistance and facilitation of plant growth. Indeed, some AHLs trigger the formation of adventitious roots due to hydrogen peroxide (H_2_O_2_) and nitric oxide (NO) dependent cyclic GMP signaling in mung bean. These two reactive oxygen are known for their effects of induce systemic acquired resistance [95,96].

### 5.2. Colonization and By-Passing of Plant Defense

Bacterial colonization depends on two distinct processes; bacterial adhesion and biofilm formation in the root or on the leaves [97]. The attachment of bacteria is an essential step for efficient colonization and is bacteria dependent. Obviously, the components of the bacterial cell surface play a significant role in the early stage of adhesion and colonization [98]. First, by means of its flagella, pili, or substances such as the exopolysaccharide (EPS) the bacteria cell moves to the beneficial position and overcome the energy barrier and bind to the plant surface. Thus, an important step in the colonization of endophytic bacteria is the formation of a biofilm that acts both as a boundary and a protective physical barrier. The biofilm formation process has been largely studied in the Gram-positive *Bacillus subtilis*. It is dependent on a large change in gene expression and particularly the transcriptional factor Spo0A, sigma-H and AbrB [99].

After establishing themselves in the interface with the plant, bacteria must move to find the best point of penetration via twitching which is under the control of two loci pilT and pilA essential for the formation and the retraction of the pilus [72,100,101]. Then, bacteria can migrate into the plant by employing different mechanisms that can be categorized into two strategies: passive and active colonization. In the rhizosphere, bacteria can passively colonize the plant interior through root wounds but also through primary and lateral root cracks [72]. They can also penetrate through natural openings on leaves and young stems, the cell wall junction like stomata, but also in hydathodes and stomatal pits or lenticels, which usually are present in the periderm of stems and germinating radicles [102]. Plant-associated bacteria such as *Burkholeria* sp., *Azoarcus* sp., *Bacillus* sp. or *Streptomyces* sp. are capable of secreting cell-wall degrading enzymes (CWDEs) that are active against the main plant cell wall components namely cellulose, hemicellulose and pectins [103,104,105]. These CWDEs allow bacteria to colonize the plant via an active form, or when they have colonized passively, continue to survive and proliferate into the host. Pectinase was demonstrated for example to be an essential enzyme for the *Azoarcus* sp. strain BH72 colonization inside rice roots [106]. Once they are situated in the host, bacterial colonization can be restricted to the specific tissue level or systematically throughout the plant. The movement of endophytes within the plant is supported by bacterial flagella, pili, and the plant transpiration stream. Furthermore, the perforated plates in the xylem vessel allow bacteria to pass through large pores without requiring specific CWDEs. Conversely, migration along intercellular spaces required the secretion of active CWDEs [100,107,108].

As they reside inside the plant, endophytic bacteria may have an advantage over rhizosphere-colonized bacteria. They are protected from environmental variations, can have access to a continuous supply of nutrients and have protection against competitive microorganisms [109]. Beneficial bacteria secrete and transport effectors proteins to the host via various kinds of transporter systems. Generally, the type I and II secretions systems are present in several beneficial endophytes. Two other types of secretion systems have been identified namely type V and VI. Type V is an autotransporter of endophytes whereas type VI (T6SSs) is a determinant in plant-microbe interactions [110]. These secretion systems allow bacteria to establish a prolonged and intimate mutualistic interaction with their host. Furthermore, to pass through the first layer of plant defense, bacteria secrete different molecules known as MAMPs which activate defensive reactions. However, some bacteria can protect themselves from these attacks by producing enzymes. In case of oxidative burst, endophytes can also produce dehydrogenases, synthases and hydratases such as superoxide dismutases (SOD), catalases (CatA), peroxidases (POD), or glutathione-S-transferases (GSTs) which prevent these beneficial bacteria from the ROS [111,112,113,114]. Another mechanism employed by bacteria to minimize the stimulation of the host’s immune system is the production of lower levels of cell-wall degrading enzymes. This pathway can act as a signal to specify to the plant that the bacteria present are beneficial [100]. Endophytic bacteria can induce phenotypic variation strategy as an adaptive process in which bacteria exhibit frequent and often reversible genetic modifications resulting in a reversible switch between colonies with different morphology [115]. Some bacteria have also co-evolved with the plant resulting in an abolishment of immune activation of the plant [116]. Thus, *P. putida* is supposed to be able to secrete AprA, which degrades flagellin monomers preventing immune recognition of flagellin in plant. This mechanism was revealed in the colonization process of this bacterium in barley roots [116]. Finally, similar to pathogens, some beneficial endophytes can modulate the expression and the concentration of the main phytohormones to promote host invasion like the PIIN_08944 effector of bacterium *P. indica*, which suppresses the expression of flg22-induced SA in *Arabidopsis thaliana* [117,118,119].

## 6. Beneficial Bacteria Implicated in Plant Defense

Beneficial bacteria present at the interface with the plant improve plant health through several mechanisms which may be direct or indirect [114,120]. In a direct approach, bacteria can promote plant growth by facilitating nutrients uptake and by reducing the effects of pathogens through competition for space and nutrients or by enzyme secretions to mediate antibiosis. On the other hand, the Induced Systemic Resistance (ISR), is an indirect and vital mechanism that explains a part of the disease suppression. Induced systemic resistance enhances the plant defense against many pathogens without directly activating important defenses while limiting the use of chemical fungicides [82,114]. These mechanisms will be explained in the context of wheat and barley defense against their pathogens (Table 2 and Table 3).

### 6.1. Induction of Plant Defense Mechanisms

#### 6.1.1. SAR and ISR Pathways

Numerous studies have demonstrated the capacity of PGPRs to improve plant health by enhancing defense against a broad range of pathogens. When PGPRs encounter the pathogen, bacteria can induce a systemic resistance known as systemic acquired resistance (SAR), which has been associated with an enhanced level of endogenous SA. Beneficial bacteria can also induce a plant reaction named ISR which is regulated by jasmonic acid/ethylene (JA/ET)-dependent signaling pathways [114,167]. The role of JA and ET in the regulation of the ISR pathway was extensively studied in *Arabidopsis* with *Pseudomonas fluorescens, Pseudomonas protegens, Serratia marcescens* and *Paenibacillus polymyxa* but also in with some agronomical crops such as tomato and cereals [114,167,168,169,170,171,172]. This mechanism is based on the activation of the potentiation of plant genes defense resulting in a faster or stronger response upon pathogen attack. During this event, a quick H_2_O_2_ accumulation and callose deposition have been observed suggesting that ISR activates the first steps of plant protection. Jasmonic acid is synthesized from α-linolenic acid (LnA) originating from chloroplast galactolipids. Jasmonic acid is under the control of a positive-feedback regulatory system which includes the SCFCOI1 complex and JAZ repressor proteins as key components in the signal transduction [76,81]. Ethylene synthesis begins with the transformation of methionine to s-adenosylmethionine (SAM). Then, it is converted into 1-aminocyclopropane-1-carboxylic acid (ACC) by ACC synthase (ACS) and 5′-methylthioadenosine (MTA) which will be recycled to L-methionine (the immediate precursor of SAM) allowing the stabilization of L-methionine levels during fairly high rates of ethylene production [69]. Finally, ET is synthesized from aminocyclopropane-1-carboxylate (ACC) via the catalysis of a second enzyme, ACC oxidase (ACO) [69,78]. Jasmonic acid is converted into the active form JA-lIe (JA-isoleucine) by JARI. Then, JA-Ile is recognized and degrades a domain of the JAZ (Jasmonate ZIM) protein, which is a transcriptional suppressor of genes dependent on JA, through the 26s proteasome. Thus, the JAZ degradation allows the activation of JA TF as ERF1 and MYC2 that induces JA-dependent gene expression such as vsp2 and pdf1.2 [114,167,173]. Furthermore, the role of MYC2 in the interaction between *H. parasitica* and *Pseudomonas fluorescens* WCS417r highlighted the important role of MYC2 as a regulator of ABA-dependent defenses which regulate a part of the primed defense, particularly the deposition of callose-rich papillae at the sites of attempted spore penetration. This shows that the activation of MYC2 modulates the expression of JA-responsive transcription factor genes, and thus indirectly affecting the expression of numerous JA-responsive genes [174,175,176,177]. Some studies have succeeded in showing a direct relationship between the reduction of the impact of certain pathogens and the stimulation of plant ISR by beneficial bacteria. For example, *Paenibacillus* sp. strain B2 was demonstrated to significantly reduce the symptoms caused by *S. tritici* by inducing an overexpression of different plant genes involved in defense and cell rescue [151]. In the same way, Petti et al. (2010) [178] carried out one of the first transcriptomic analyses on barley to understand the impact of the *Pseudomonas fluorescens* strain MKB158 to protect from Fusarium head blight. They discovered a regulation on the JA/SA and ET pathways and an induction of long-distance signaling during SAR.

The SAR and ISR pathways are two distinct modes of defense with one particularly efficient against biotrophic and hemibiotrophic pathogens and the other against necrotrophic pathogens respectively. Generally, the JA/ET and SAR pathways act antagonistically; at a high level, SAR often antagonizes the ISR reaction by inhibiting JA signaling. Furthermore, ET acts positively on the expression of the ERF branch of the JA pathway whereas it suppresses the MYC branch which results in a prioritization of the ISR reaction. However, because of the constant pressure on plants by different pathogen lifestyles, there exists a SA-JA/ET cross talk which can be a potent mechanism to prioritize one pathway depending on the pathogen. Mur et al. (2006) [179] observed that the concentration of applied JA and SA can directly affect the response of the plant. In *Arabidopsis thaliana*, at low concentrations, the three main phytohormones act synergically on the JA and SA-responsive genes PDF1.2 and PR-1, respectively, whereas, at high concentrations, SA suppresses the JA pathway. *NPR1* is an essential component in the SA-JA/ET crosstalk, although its role appears to be different. In SAR signaling, *NPR1* is connected to a function in the nucleus while in the JA/ET signaling, it is associated with cytosolic function. Some studies indicate that SAR and ISR do not compete for the same pool of *NPR1* [114,167]. Nie et al. (2017) [180] demonstrated that *B. cereus* AR165 induces plant resistance against *Pseudomonas syringae* DC3000 in *Arabidopsis thaliana*. The ISR pathway is dependent on SA and *NPR1* but not JA/ET. They also showed that AR156 is effective against *B. cinerea* but in this case, via the JA-ET pathway and *NPR1*. Thus, this study highlighted that *NPR1* is necessary in both cases and the same bacteria can induce two types of reactions.

#### 6.1.2. Priming Effect

Following the perception of stimulus, plants can improve their resistance capacity by using a long-term sensitization which could allow them to react faster and more strongly. This phenomenon called priming is an adaptative strategy that includes the SAR pathway, induced by necrotizing pathogens, but it also includes ISR, activated by beneficial bacteria [114]. Priming is defined as “an induced, triggered or activated state in which the plant is initially treated with the prime inducing agent that enhances its resistance against secondary stresses” [174]. This supposed that the enhanced capacity of plants defense is not linked with direct activation of defensive genes but a stronger activation of basal defense and an accumulation of dormant protein kinases, which would require a secondary post-translational modification [181]. Cantoro et al. (2020) demonstrated an induced strengthening of epidermal cell wall after inoculation of *B. velezensis* RC 218 in the wheat tissues surrounding the *F. graminearum* infection site. The same strain was also studied for its capacity to induce plant defense in susceptible wheat cultivars by preventing an increase of pathogen-related hormones caused by the infection of F. *graminearum* on wheat spikes [126]. These studies highlighted the protective effect of the bacterium to limit the proliferation of the pathogen with activation of basal defense. Hypotheses regarding priming mechanisms have been demonstrated. The process can be divided into three main phases: (i) stimulus perception or priming phase, (ii) secondary stimulus challenges or primed state after challenge, and (iii) trans-generational phase or inherited state from primed parents [182]. During the first step of the perception, the cytosolic calcium increases trigger ion fluxes across the membrane to lead membrane depolarization. Depolarization is followed by a production of reactive oxygen species (ROS) which is crucial for the induction of defense. Furthermore, structural modifications occur during priming with callose deposition and infiltration of phenolic compounds to create an impermeable barrier to pathogen penetration [183]. In *Arabidopsis thaliana*, the SAR activator benzo (1,2,3) thiadiazole-7-carbothioic acid S-methyl ester was correlated with an accumulation of mRNA transcripts and inactive proteins of mitogen-activated protein kinase 3 (MAPK3) and MAPK6 and not a direct activation of *PR* [184]. The MAPK proteins are three-tiered signaling kinase modules that are receptors implicated in cellular signal amplification. The increase of MAPK level is associated with an enhancement of *PR1* and PAL1 defense genes [181]. Thus, in the dormant phase, these proteins are a part of the mechanism explaining the prolonged defense priming. Furthermore, priming is also driven by the recognition of the bacteria by a plant receptor. Among these receptors, FLS2 is a leucine-rich repeat receptor kinase (LRR-RK) of the conserved N-terminal amino acid epitope flg22 in the bacterial MAMP flagellin which interacts with its signaling partner, BRI1-ASSOCIATED RECEPTOR KINASE1 (BAK1). Their interaction induces phosphorylation of both but also an accumulation of ROS and activation of MAPK cascade composed of MEKK1, MKK4/5, and MAPK3/6 [185]. The authors demonstrated that after treatment with benzothiadiazole in *Arabidopsis thaliana*, the levels of FLS2 and BAK1 increased with an enhancement of plant response to flg22. This finding suggests that priming increases the biosynthesis and the secretion of *PRRs* to the plasma membrane allowing the plant to respond more specifically and more quickly to MAMPs and PAMPs even at low doses.

### 6.2. Direct Antagonism

#### 6.2.1. Space and Nutrients Competition and Plant Health

While beneficial bacteria are situated in the rhizosphere, they can directly interact with other microorganisms and compete with plant pathogens for space and nutrients. Competition for nutrients and space is considered as a means to limit the development of pathogens via the reduction of the number of habitable sites and thus, the inhibition of the germination of fungal spores in the soil [136]. When the antagonist is present in sufficient quantity at the right place and at the right time, it can reduce the fungal pressure. Besides, space competition has been demonstrated in the soil but also at the plant level [186]. Beneficial bacteria can colonize the plant root through the formation of biofilms and can also penetrate directly into the plant, hence limiting the pathogen. For example, *Bacillus cereus* is supposed to protect wheat crops against *Septoria tritici* by excluding the pathogen from the stomata and substomatal chambers [121]. Concerning the competition for nutrients like carbon, nitrogen or iron, this method can be considered as a common way to limit the growth of other microorganisms even if demonstrating its actual effect at field scale is difficult. A previous study showed that *Pseudomonas* sp. AS 64.4 reduced the Fusarium head blight (FHB) disease incidence by metabolizing chlorine, which is an essential nutrient found in wheat flower tissues, because chlorine is used as a source of carbon for the pathogen [154]. When iron becomes limiting, bacteria can produce a range of iron-chelating compounds known as siderophores which have a strong affinity with iron [187,188]. This strategy to take up available iron is a competitive advantage for the development of beneficial bacteria as it reduces the availability of the element for pathogens. Thus, similar to *Streptomyces* spp. and *Pseudomonas mediterranea* can inhibit different wheat pathogens including *F. oxysporum*, *F. moniliforme*, *P. oryzae*, and *M. phaseolina* [189]. The inhibition of pathogen growth or the inhibition of metabolic activity were also reported in different crop systems with *Kosakonia radicincitans* against *Penicillium expansum*, *Botrytis cinerea*, *Rhizopus* sp., *Alternaria* sp. and *Cladosporium cladosporioides* or with *Pseudomonas* strains GRP3A against *Colletotrichum dematium*, *Rhizoctonia solani* and *Sclerotium rolfsi* [190,191].

Furthermore, beneficial bacteria may also protect the plant by affecting the plant growth and development, and by facilitating nutrients acquisition. Certainly, plants need necessary elements like iron, nitrogen, and phosphorous to grow properly and optimize their yield and, at the same time, resist biotic stresses. However, these elements are rarely available in the environment because of their presence in an unassimilable form [120]. Among the essential nutrients, iron is essential for the growth, metabolism, and survival of the plant although the free form is rare in the environment because of the oxidation converting much of soluble iron into insoluble ferric oxides and oxyhydroxide. Wheat and barley plants have different strategies to acquire iron: the first one is the acidification of the rhizosphere followed by reduction of Fe^3+^ ions by membrane-bound Fe^(III)−^ chelate reductase and then the uptake of Fe^2+^ into the cell. The second involved the secretion of phytosiderophore to solubilize the bound iron which is then transported into the cell in membrane protein [192]. Microbial siderophores are mainly used by the plant for iron uptake from the soil considering the fact that the plant can produce siderophores and other molecules negatively charged that can bind iron, but their affinity is must lower than microbial siderophore. Siderophores chelate ferric iron to form ferric siderophore complexes that are transported into the bacteria cell where ferric iron is released from the siderophore and reduced to Fe^2+^ to be utilized for metabolic processes [193,194,195]. Several bacteria have already been identified for their biocontrol efficacity against wheat pathogens. An in vitro essay from Lounaci et al. to analyze wheat protection from *F. graminearum* suggested that the production of siderophore by the bacterium *Paenibacillus polymyxa* SGK2 was one of the main mechanisms involved in the fungal inhibition [149]. Likewise, an assay realized with siderophore *Pseudomonas putida* strain BK8661 mutants revealed that antibiotic and siderophores production have a suppressive effect on *Z. tritici*. Furthermore, nitrogen is the most important element because it is the main constituent of amino acids, proteins and nucleic acids, constituting DNA and RNA but it must be reduced to ammonia before it can be metabolized by plants [196]. Some strains like *Bradyrhizobium japonicum* can convert H_2_ from the atmosphere into H^+^ via hydrogenase and produce ATP that can be used to fix more nitrogen. However, this particularity is not commonly found in the natural microbiome. Even if the fixation of the nitrogen costs energy to the bacteria, some studies revealed a direct impact of these bacteria on plant growth, especially during plant abiotic stress [197]. Thus, even if the promotion of plants can be efficient under abiotic stress, they can also be beneficial to control the cereals pathogens by making plants more vigorous and more resistant.

#### 6.2.2. Secretion of Metabolites

##### Enzymes

Another mode of action employed by beneficial bacteria to inhibit pathogen proliferation is the direct confrontation and the secretion of antifungal compounds. Mycoparasitism is one of the most important antagonism involving direct physical contact with bacterial enzymes produced against the fungus thereby causing digestion of the host cell [198,199]. However, even if this mechanism is usual for some fungus such as *Trichoderma* spp., bacteria from *Bacillus* spp. [22,121,131,134], *Pseudomonas* spp. [154,155], *Streptomyces* spp. [126,137,141,142], *Burkholderia* spp. [200,201], and *Lactobacillus* spp. [148], routinely secrete antifungal enzymes, secondary metabolites or cell wall degrading enzymes. Among these antifungal enzymes, bacteria secrete β-1,3 and β-1,4 glucanases, chitinases, lipases and proteases. Moreover, these enzymes degrade the main structural component of pathogenic fungal cell walls namely chitin, β-1,3-glucan and protein. The β-1,3 glucanases hydrolyze the O-glycosidic linkages of β-1,4 glucan chains by sequentially cleaving glucose residues from the non-reducing end and by cleaving β-linkages at random sites oligosaccharides [202]. The action of enzymes against *F. graminearum* was demonstrated by He et al. (2009) [150] with *Paenibacillus polymyxa* W1-14-3 and C1-8-b which produce cell-wall degrading enzymes to disrupt the integrity of the pathogen. *Bacillus subtilis* SG6 could also induce the cell wall degradation enzyme of *F. graminearum* by chitinase secretion [203]. The compound 2,4-diacetylphloroglucinol produced by *P. fluorescens* PFM2 may also be implicated in the natural antagonism between the beneficial strain and *Z. tritici.* In addition to their effect on the integrity of the cell wall, bacteria can also affect the virulence of the pathogen as it was the case with *Bacillus* sp. 240B1 which is capable of enzymatic inactivation of autoinducers Ais (N-acylhomoserine lactones) by releasing AiiA enzyme. The AiiA enzyme is a lactonase that inactivates acyl-homoserine-lactone molecules which regulate the expression of virulence genes of *Pseudomonas solanacearum* [204,205].

##### Volatiles Compounds

Furthermore, beneficial bacteria can produce a wide variety of antibiotics and secondary metabolites which are differentially bioactive. Among metabolites, volatile compounds (VOCs) can inhibit the development or the germination of pathogens or activate plant defense mechanisms via induction of ISR. They are small odorous compounds (<C15) with low molecular mass (<300 Da), high vapor pressure, low boiling point, and a lipophilic moiety [206]. They are divided into different chemical classes including alcohols, alkenes, benzenoids, ketones, pyrazines, sulfides, and terpenes. The VOC production is dependent on the environmental conditions (temperature, oxygen availability, pH among others) and the growth stage of the bacteria and follows the GacS/GacA two-component regulatory system for many bacteria. The VOCs are considered to be a major key regulator system in the induction of ISR and induce the priming effect but also affect the plant’s secondary metabolite production and influence plant development via the modification of the hormone pathway. Thus, the VOC produced by *B. subtilis* GB03, named 2,3-butanediol, is known to be one of the major VOCs produced by *Bacillus*, and plays a critical role in the induction of systemic resistance against *P. carotovorum*. *Bacillus* secretes alcohols, such as 3-methyl-1-butanol, 2-methyl-1-butanol and butane-1-methoxy-3-methyl, which share a similar functional motif to that of 2,3-butanediol [207]. Li et al., (2015) [208] showed that emitted VOCs (mostly ketones and alcohols) obtained from eight *Bacillus* strains demonstrated 56–82% inhibition against the mycelial growth of *Fusarium solani*. The VOCs also inhibit pathogen growth by direct confrontation. Interestingly, a direct confrontation of *Streptomyces salmonis* PSRDC-09 with *C. gloeosporioides* PSU-03 led to the production of l-linalool that induced irregular distortions in the fungal hyphae [209]. A study of wheat protection against *Z. tritici* also demonstrated due to a dual plate culture assay the effect of VOC production by *B. megaterium* MKB135 on the pathogen germination [135]. Likewise, *Streptomyces albulus* NJZJSA2 produced 4-methoxystyrene, 2- pentylfuran, and anisole and successfully suppressed *Sclerotinia sclerotiorum* and *Fusarium oxysporum* [210].

##### Secondary Metabolites

Beneficial bacteria belonging to the *Bacillus, Pseudomonas, Burkholderia*, and *Streptomyces* genera produce a broad arsenal of secondary metabolites which are involved in direct antibiosis and induction of systemic resistance against plant-pathogens [211,212,213]. *Pseudomonas* strains produce several molecules involved in plant pathogen interactions including phenazines, pyoluteorins, pyrrolnitrins, 2,4-diacetylphloroglucinol and lipopeptides. On the other hand, *Bacillus* strains directly suppress plant pathogens by producing lipopeptides (iturin, surfactin and fengycin), siderophores, polyketides and oligopeptides among others [214].

Secondary metabolites of *Pseudomonas* have been implicated in the biocontrol of several plant pathogens [215,216]. *Pseudomonas* strains are interesting for application because of the enormous diversity of metabolites produced and for their good adaption to multiple environments. Towards the biocontrol of cereal pathogens, phenazine-1-carboxylic acid, produced by *Pseudomonas fluorescens* and *P. aureofaciens* suppressed *Gaeumannomyces graminis var. tritici* on wheat [217,218]. In another study, 2,4-diacetylphloroglucinol was shown to suppress the same pathogen on wheat [219,220]. Furthermore, pyocyanin derived from *P. aeruginosa* was effective against the wheat pathogen *Septoria tritici* [155,221,222]. In multiple studies, the pyrrolnitrin antibiotic from *P. fluorescens* effectively controlled *Pyrenophora tritici-repens* on wheat [223,224]. Several lipopeptide-producing pseudomonads have been isolated from the wheat rhizosphere [225] and show antagonism against soil-borne plant pathogens. However, to the best of our knowledge, none of these strains or their corresponding lipopeptides have been tested for efficacy against the main foliar pathogens of wheat and barley.

*Bacillus* strains are largely studied because of their capacities to suppress and inhibit plant pathogens, particularly through metabolites production. *Bacillus* strains can devote 4–5% of their genome to synthesize secondary metabolites [123]. The antimicrobial peptides are synthesized ribosomally or non-ribosomally by non-ribosomal peptide synthetases (NRPSs) and act directly in the suppression of pathogens. Bacteriocins are an example of ribosomally synthesized peptides including lantibiotics such as amylolysin, mersacidin, subtilin among several others [226]. Non-ribosomal peptides correspond to the unusual peptides dipeptides and oligopeptides, the cyclic lipopeptides (CLPs) and the polyketides (Pks). The dipeptides and oligopeptides include bacilysin, rhizocticin, chlorotetain, and bacitracin. Bacillaene, difficidin and macrolactin are the three main types of PKs [227]. The antifungal proprieties of the cyclo-lipopeptides (CLPs) have been widely demonstrated for many diseases. Thus, in contrast with *Pseudomonas* metabolites, the *Bacillus* LPs have been well studied against a wide range of cereal pathogens.

Lipopeptides (LPs) are described for their antagonism function, mobility and attachment to surface [228]. In general, LPs have been shown to have a strong protective effect against several pathogens on host plants. They are highly resistant to the environmental conditions and the attack by peptidases, proteinases, or oxidases. Their biosurfactant proprieties enable them to interfere with the cell wall membrane permeability in a dose-dependent manner which causes inhibition of the cell wall or the formation of spores to the pathogen. The LPs are known to penetrate the fungi cell membranes to form ion channels and cause the membrane osmotic imbalance causing cell death [212,228].

Although *Bacillus* produce several antibiotics according to strains, the main CLPs excreted by the bacterial strains can be classified into three main families, surfactins, iturins and fengycines, according to their amino acid sequence, the structure within the peptide ring, and the type and/or attachment of the fatty acyl chain [229]. They are synthesized by modular mega-enzymes called non-ribosomal peptide synthetases (NRPSs) [211]. The peptide part is hydrophilic while the lipid part is lipophilic thus resulting in amphiphilic molecules. This particularity has a direct consequence on the physico-chemical properties of these molecules and their biological activity as is the case for surfactins which are potent surfactants. Within each family, there are isoforms, differentiated by the nature of amino acids in the cycle, and homologs, which vary according to length and lipid chain isomeric [230]. The iturin family includes bacillomycin, iturin and mycosubtilin. They are cyclic lipoheptapeptides bound by a residue of ß-amino acid. It is known to disturb the cytoplasmic membrane causing leaching of K+ ions and other constituents involving cell death.

The annotation of the complete genome of *Bacillus amyloliquefaciens* AS 43.3 revealed nine gene clusters encoding secondary metabolites associated with the control of *Fusarium graminearum* [231]. Five of these clusters were identified as non-ribosomal peptide synthetases encoding three lipopeptides (surfactin, iturin, and fengycin), one siderophore (bacillibactin), and one antibiotic (bacilysin). *Bacillus amyloliquefaciens* S76-3 displayed a strong antifungal activity against *Fusarium graminearum* by producing iturin A and plipastatin A which caused leakage of fungi cellular contents and vacuolation [131]. The most active form of iturin, mycosubtilin, interacts with the phospholipid and sterols, especially with the acyl chains of the phospholipid and alcohol group of cholesterol in the membranes [123,131]. Fengycins are the other cyclic-lipopeptides family with antifungal activity like the fengycin produced by *Bacillus subtilis* strain IB which exhibit strong activity against *F. graminearum* due to the production of this metabolite [128,232]. Fengycin has a broad spectrum of action but is particularly effective against filamentous fungi. Like iturins, fengycins interact with the membrane by inserting the fatty acyl chain with the cyclic peptide region from the membrane leading to an increased thickness of the bilayer [229]. At high fengycin concentration, the cyclic peptide sections of the lipopeptides can increase the positive curvature of the membrane due to the shape of the compound leading to the formation of disk-like micelles of membrane phospholipids supported around the edges by the amphiphilic fengycins. When the concentrations are high, the pore formation in the membrane can lead to complete solubilization of the membrane into micelles as it was demonstrated by Deleu et al. (2005) [232]. Lastly, the surfactin family is composed of variants with heptapeptides interlinked with a β-hydroxy fatty acid to form a cyclic lactone ring structure [233]. Like fengycin, in membrane model, surfactin can insert the fatty acyl chain into the membrane but, in this specific case, the insertion is independent of the phase of the fatty acyl chain within the membrane and can be divided into two steps: peptide insertion into the membrane and electrostatic repulsion between charges borne by lipid headgroups and the negatively charged SF amino acid [234]. However, surfactin has no direct effect on fungi but seems to act synergically with iturin A activity [235]. The study of Cawoy et al. (2014) [236] revealed that surfactin and fengycin also induce the ISR mechanism in the tobacco plant. A strong correlation was found between the plant defense mechanism and the concentration of surfactin secreted by the bacteria. Cyclic lipopeptides were shown to be a non-volatile elicitor by stimulating the oxylipin pathway. Moreover, the coproduction of chitinases, fengycin and surfactins significantly reduced the development of *Fusarium graminearum* and DON production on wheat [203]. Surfactin is also involved in the attachment and aggregation of microcolonies in the formation of biofilm. This property is a key determinant in the control of some pathogens. The surfactin produced by *B. subtilis* 6051 participates in the formation of a stable biofilm on *Arabidopsis thaliana* roots. The deletion of the surfactin synthase gene in the strain decreases the rate of colonization of the bacteria and the biocontrol activity against *P. syringae* [237]. In this way, surfactin can be a good inhibitor of pathogen adhesion and biofilm formation to prevent them from infecting the host. Surfactin and iturin can also modulate the mobility of bacteria facilitating the colonization of the bacteria [238,239].

#### 6.2.3. Limitation of Mycotoxins Production

The contamination of plants and thus food by mycotoxins is a significant cereal production problem. Mycotoxins are considered as major food safety issues due to their highly toxicity and their carcinogenic properties on animals and humans. Their regulation is traced by the International Agency for Research on Cancer and is subject to regulations with maximum residue levels. Aflatoxins, ochratoxin A and *Fusarium* toxins are the main mycotoxins that contaminate wheat and barley grains. Aflatoxins are mainly produced by *Aspergillus flavus* and *Aspergillus parasiticus*, while ochratoxins are a group produced by *Aspergillus ochraceus*, *Penicillium verrucosum*, and other *Penicillium* species. Both can contaminate processed products and animal-derived products which can lead to health problems that can be lethal in extreme cases [240,241].

Among *Fusarium* toxins, the zearalenone (ZEA) and deoxynivalenol (DON) produced by *Fusarium* species especially *F. culmorum* and *F. graminearum* are also considered as metabolites of particular concern to food and regulation agencies [150]. During the first step of the infection when the pathogen survives biotrophically, DON is assumed to be unimportant. However, at high concentration, DON in the plant cell promotes the synthesis of H_2_O_2_, resulting in the inhibition of defense-related responses and cell death promoting pathogen necrotrophic growth [30,242]. Currently, several methods are used to limit the risk of contamination of these mycotoxins but they are costly for a limiting efficacity [243]. Thereby, in recent years, the study of microorganisms and especially beneficial bacteria able to degrade mycotoxins to non or less toxic compounds appear promising [244]. In addition to the direct effects of the beneficial bacteria, their ability to reduce or to alter the production of mycotoxins by the pathogen can be an essential step in the reduction of the pathogen severity. Among these bacteria, several *Rhodococcus*, *Pseudomonas* or *Bacillus* species are instrumental in the degradation of ZEA. Altalhi et al. (2007) [245] demonstrated that *Pseudomonas putida* ZEA-1 was able to use ZEA as a carbon source by transforming the toxin into products with less or no toxicity. Moreover, *B. licheniformis* CK1 [246], *B. subtilis* [247], *B. subtilis* ANSB01G showed potential to degrade ZEA. In addition, the use of DON-degrading bacterium has been the subject of several studies [137,160,203,248,249,250]. Shima et al. [250] showed that the bacterial isolate E3-39, obtained from a mixed culture, had the capability to oxidize the 3-OH group of DON to generate 3-keto-4-deoxynivalenol (3-keto-DON) leading to less than one-tenth immunosuppressive toxicity relative to deoxynivalenol. Similarly, the bacteria *Devosia* mutants 17-2-E-8 were able to convert DON into a major and minor product, named 3-epi-DON and 3-keto-DON, respectively [249]. In a separate study, 13 isolates belonging to the genus *Nocardioides* and *Devosia* were obtained from field soils and wheat leaves and were all able to degrade 100 μg mL^−1^ DON [160,248].

## 7. Conclusions and Perspectives

In this review, we describe the main diseases affecting wheat and barley production and their causative pathogens. Furthermore, we discussed the current control measures being applied while highlighting the emerging potentials of biological control. By summarizing the general insights into molecular actors involved in plant-pathogen interactions, we show to an extent, how beneficial bacteria are implicated in plant defense against wheat and barley diseases.

For the control of wheat and barley diseases, previous research has focused predominantly on chemical methods, prophylactic strategies, and genetic selection. More recently, there has been a surge in the exploration of biological control methods albeit, with limited in-depth studies. Biological control is especially worthy of exploration in view of current trends to limit the use of environmental pollution due to pesticides use. Thus, we have summarized information on beneficial bacteria with demonstrated antifungal activity against pathogens of wheat and barley, including known modes of action while identifying potential areas for in-depth research. First, it appears that the origin of beneficial microbes could count towards their selective mode of action. Specifically, *Pseudomonas* strains derived from the wheat/barley phyllosphere or kernels/anthers suppressed target wheat and barley pathogens via production of hydrolytic enzymes or antibiotics such as DAPG, HCN, siderophores and phenazines (pyocyanin, phenazine-l-carboxylic acid and phenazine-1-carboxamide) [155,158,160]. Thus, disease suppression by these metabolites was efficient both through direct contact with the pathogen and via induced systemic resistance. To the best of our knowledge, most of the beneficial bacteria with efficacy against barley pathogens originated from the soil or maize/sorghum/crawberry rhizosphere. Exceptionally, seeds treatment with the crawberry rhizosphere isolate *P. chlororaphis* MA 342 (Cedomon, Cerall) has shown good disease control effects against *P. teres* in barley and *S. nodorum* in greenhouse experiments [157,163].

In contrast, numerous foliar- and soil-derived *Bacillus* species have been well studied in the control of wheat pathogens. This genus mediates direct antagonism or ISR via competition for space and nutrients or mainly through production of secondary metabolites which are either synthesized (non-) ribosomally. Such non-ribosomal peptides include mycosubtilin, surfactin, fengycin, iturin, plipastatin and bacillomycin among others [122,130,131,138]. It appears that for most of the *Bacillus* strains tested, the mode of action is suggested and not shown experimentally, which should be the focus for future studies [121,136,141]. In a very recent study, rhamnolipids (synthesized by species in *Pseudomonas* and *Burkholderia* genera), were shown to protect wheat against *Z. tritici* mainly through direct antifungal activity and without major impact on leaf physiology [251]. Such in-depth studies that explore the use of antimicrobial molecules accompanied by gene expression and metabolic profiling studies will expound our understanding of cereal-bacteria-metabolites-based research thereby contributing to increased barley and wheat production.

During the past decade, beneficial bacteria have emerged as promising alternatives compared to the use of chemical pesticides. However, the commercialization of strains efficient in greenhouses and fields is still a long way off for obvious reasons. Bacteria are very sensitive to their environment such as the temperature and humidity fluctuation, competition with other species, UV and the plant host, among others [252]. In this review, we have showed that the biocontrol commercial products available against cereal pathogens are not the main research topic of companies which focus especially on *Trichoderma*, *Pseudomonas* or *Streptomyces*. Noticeably, no *Bacillus*-based products are available. Clearly, a lot more needs to be done to translate in vitro and greenhouse-based research to practical solutions towards sustainable protection of wheat and barley pathogens. Factors to be considered include specific plant and pathogen lifestyle, the timing of biocontrol agent application, the persistence on the plant and host-specific responses to the biocontrol agent.

## Figures and Tables

**Table 1:** Commercial bacterium-based products against barley and wheat pathogens.

Products Name	Company	Beneficial Microbes	Pathogen	Targeted Crop	Mode of Action for Biocontrol
Polyversum^®^	De Sangosse	*Pythium oligandrum* M1	*Fusarium* and *Sclerotina*	Wheat, barley, and colza	- Space and nutrients competition - Hyperparasitism - Metabolites
Mycostop^®^	Lallemend	*Streptomyces* sp. K61	*Fusarium*	Wheat, corn, barley, sugar-beet, and tomato	- Space and nutrients competition - Hyperparasitism - Metabolites
Inatreq™ Active^®^	Corteva	Fenpicoxamid from fermentation broths of the *Streptomyces* sp. 517–02	*Septoria*	Wheat	- Inhibition of mitochondrial complex III via binding to the Q_i_ ubiquinone binding site
Cerall, Cedomon^®^	Koppert	*Pseudomonas chlororaphis* MA342	*Fusarium* spp., *Septoria*, wheat bunt and *Drechslera teres*	Wheat, barley, triticale, and rye	- Space and nutrients competition - Metabolites - ISR - Plant Growth Promotion

**Table 2:** Beneficial bacteria which demonstrated protection against main wheat pathogens. n/a: not applicable.

Strains	Origin	Pathogen	Biostimulation	Mode of Action for Biocontrol	Methodology	References
*Bacillus subtilis* ATCC 10783; *B. cereus* ATCC 11778; *B. licheniformis* NRRLB-510; *B. pumilus* ATCC 7061; *Brevibacillus laterosporus* BLA170; *Paenibacillus polymyxa* NA	Soil	*Zymoseptoria tritici, Pyrenophora tritici-repentis, Cochliobolus sativus, Alternaria triticimaculans*	n/a	n/a	In vitro and greenhouse (leaves)	[121]
*B. subtilis* BBG125, BBG131, Bs2504	ProBioGEM, Centre Wallon de Biologie Industrielle	*Z. tritici*	n/a	Lipopeptides (LPs): mycosubtilin, surfactin, fengycin	In vitro and greenhouse (leaves)	[122]
*B. velezensis* RC 218	Wheat anthers	*Fusarium graminearum*	n/a	Ericin lantiobiotic Plant phytohormone modulation (jasmonic and salicylic acid) ISR cell wall thickening preventing cell plasmolysis and collapse	Field and greenhouse (spikes)	[123,124,125,126]
*B. velezensis* LM2303	Wild yak	*F. graminearum*	n/a	n/a	In silico and field (spikes)	[127]
*B. subtilis* IB	Soil	*F. graminearum*	n/a	Fengycin	In vitro	[128]
*B. megaterium* BM1 and *B. subtilis* BS43, BSM0, BSM2	Wheat spikes	*F. graminearum*	n/a	Degradation of DON Metabolites	In vitro and field (spikes)	[129]
*B. subtilis* BaSu1/BaSu3, *B. amyloliquefaciens* BaAm and *Chaetomium globosum* CG1/CG2, *Phoma glomerata* PG1, *Aureobasidium proteae* AP5 and *Sarocladium kiliense* SK1/SK2	Wheat endosphere	*F. graminearum* and *F. culmorum*	n/a	Antibiosis	In vitro and greenhouse (detached wheat spikelets)	[22]
*B. amyloliquefaciens* TrigoCor	Wheat rhizosphere	*F. graminearum*	n/a	Iturin	Field and greenhouse (spikes)	[130]
*B. amyloliquefaciens* S76-3	Wheat spikes	*F. graminearum*	n/a	Iturin A and plipastatin	In vitro	[131]
*B. amyloliquefaciens* Y1	Soil	*F. graminearum*	n/a	Metabolites Cyclo D-PRO-L- VAL	In vitro	[132]
*B. amyloliquefaciens* B8 and B3	Soil	*F. graminearum* and *culmorum*	Yes	Phytohormones	In vitro and greenhouse	[133]
*B. amyloliquefaciens* BLB369, *B. subtilis* BLB277, *Pae. polymyxa* BLB267	Soil	*F. graminearum*	Yes	Supernatant (iturin and surfactin, fengycin, fusaricidin and polymyxin)	In vitro and greenhouse	[134]
*B. megaterium MKB135* *Pseudomonas fluorescens MKB21 and MKB91*	Cereal rhizospheres, leaves, grain and weeds	*Z. tritici*	Yes	Cell free surpernantant and VOC	Field and greenhouse (leaves)	[135]
*B. subtilis* strains 53 and 71, *P. fluorescens* biov1 strain 32 and *Streptomyces* sp. strain 3	Wheat kernels	*F. graminearum*	Yes	Volatiles metabolites	In vitro and greenhouse (spikes)	[136]
				Antibiotics tubercidin, phosphlactomycin and candicidin, 2,4-diacetylphloroglucinol, phenasin, fengymcine, bacillomycin		
				Phytohormone regulation		
*B. subtilis* RC 218, *S.* sp. BRC87B. and *Brevibacillus* sp. BRC263	Wheat anthers	*F. graminearum*	Yes	Space and nutrients competition	Field, greenhouse and in vitro	[125,126,137,138,139,140]
				Metabolites		
*Streptomyces.* sp. DEF09	Wheat root	*F. graminearum*	Yes	Metabolites	Field, greenhouse (spikes) and in vitro	[141]
				IAA		
*Streptomyces.* sp. BN1	Rice kernels	*F. graminearum*	n/a	n/a	In vitro and greenhouse (seeds and spikes)	[142]
*B. cereus*	Soil from wheat fields	*F. graminearum*	Yes	Dose and cultivar dependent	In vitro and greenhouse (seeds)	[143]
*B. subtilis* AS43.3/AS43.4, *Cryptococcus* sp. OH71.4 and *Cryptococcus nodaensis* OH182.9	Wheat anthers	*F. graminearum*	n/a	n/a	Field, greenhouse (spikes) and in vitro	[144,145]
Co-cultures of *B. subtilis* OH 131.1 and *Cryptococcus flavescens* OH 182.9	ARS NRRL	*F. graminearum*	n/a	Plipastatin and subtilomycin	Greenhouse (spikes)	[146]
*Lactobacillus brevis* JJ2P; *Lactobacillus reuteri* R2	Porcine gut, cheese	*Z. tritici*	n/a	Cell free supernatant (phenyllactic acid and hydroxyphenyllactic acid)	In vitro and greenhouse (leaves)	[147]
*Lactobacillus plantarum strain* 21B	Sourdough breads	*F. graminearum*	n/a	Antifungal phenyllactic acid and 4-hydroxyphenyllactic acid	In vitro	[148]
*Paenibacillus polymyxa* SGK2	Wheat rhizosphere	*F. graminearum, F. culmorum, F. verticillioides*	Yes	Competition for nutrients (iron)	In vitro and greenhouse (seeds)	[149]
*Pae. polymyxa* W1-14-3 and C1-8-b	Rhizosphere	*F. graminearum*	Yes	Inhibition of fungal germination	In vitro and greenhouse (spikes)	[150]
				glucanolytic enzyme, cellulase, mannanase xylase, chitinase and protease		
				supernatant activity (enzymatic or antibiotic activities: polymyxins, benzoic acid, fusaricidin A and antibiotic peptides)		
*Pae.* sp. B2	INRAE Dijon	*Z. tritici*	n/a	ISR	Field and greenhouse (leaves)	[151]
*P. fluorescens* LEC1	Wheat phyllosphere	*Z. tritici*	n/a	Antibiotics 1- hydroxyphenazine and chlororaphin	In vitro and greenhouse (leaves)	[152]
*P. fluorescens* PFM2	Wheat phyllosphere	*M. graminicola*	n/a	Antibiotics 2-4-diacetylphoroglucinol and phenazine-l-carboxylic acid	In vitro	[153]
*P sp.* AS 64.4	Wheat anthers	*F. graminearum*	n/a	Nutrients competition (choline metabolizing strain)	Field, greenhouse (spikes) and in vitro	[154]
*P. putida* BK8661	Wheat phyllosphere	*Z. tritici*	Yes	HCN, siderophore, antibiotics	In vitro and greenhouse (leaves)	[155]
*P. aeruginosa* LEC1	Soil	*Z. tritici*	n/a	Antibiotic (Pyocyanine) and Siderophore (pyoverdine)	In vitro and field (leaves)	[156]
*P. chlororaphis* MA 342	Craw berry rhizosphere	*Septoria nodorum*	n/a	n/a	Field (seeds)	[157]
*P. piscium* ZJU60	Wheat anthers	*F. graminearum*	n/a	Phenazine-1-carboxamide	Field and greenhouse (spikes)	[158]
*P. fluorescens* LY1-8	Wheat tissues	*F. graminearum*	n/a	Extracellular hydrolytic enzymes (protease, chitinase, cellulose, glucanase and siderophore) and antagonistic activity	Field and greenhouse (spikes)	[159]
*Devosia* sp. strain NKJ1 and *Nocardioides* spp. strains SS3 or SS4	Wheat field soil	*F. graminearum*	Yes	Degradation of DON	In vitro	[160]

Rows highlighted in grey depict the authors hypothesis for the mode of action of beneficial bacteria based on their preliminary studies.

**Table 3:** Beneficial bacteria which demonstrated protection against main barley pathogens.

Strains	Origin	Pathogen	Biostimulation	Mode of Action of Biocontrol	M&M	Source
*Pseudomonas fluorescens* MKB100 and MKB156	Cereal rhizosphere	*P. teres*	n/a	ISR	Field, greenhouse (leaves and drenching) and in vitro	[161]
				Production of antifungal compounds (2,4-DAPG and HCN)		
*Pseudomonas chlororaphis* MA 342	Craw berry rhizosphere	*D. teres*	n/a	n/a	Field and greenhouse (seeds)	[157,162,163]
		*D. graminea*				
		*U. hordei*				
*Paenibacillus polymyxa* KaI245	Sorghum rhizosphere	*Drechsclera teres f.* sp. *teres* and *Rhynchosporium commune*	Yes	Cell free supernatant	In vitro and greenhouse (leaves)	[164]
*Burkholderia* sp. strain BE25	Maize rhizosphere	*P. teres*	Yes	Induction plant genes defense	In vitro and greenhouse (leaves)	[165,166]
				Limitation of the fungus on photosynthetic and respiratory parameters		

Rows highlighted in grey depict the authors hypothesis for the mode of action of beneficial bacteria based on their preliminary studies.

## Data Availability

Not applicable.
